# Supplementary material for: Quantitative evaluation of methods to analyze motion changes in single-particle experiments
Source: Nat Commun. 2025 Jul 22;16:6749. doi: 10.1038/s41467-025-61949-x (PMC12283970; doi:10.1038/s41467-025-61949-x)
Supplement: Supplementary file 1 — Supplementary Information [file 41467_2025_61949_MOESM1_ESM.pdf]

SUPPLEMENTARY INFORMATION

Quantitative evaluation of methods to analyze motion changes in single-particle experiments

Gorka Muñoz-Gil,[1,*] Harshith Bachimanchi,[2] Jesús Pineda,[2] Benjamin Midtvedt,[2] Gabriel Fernández-Fernández,[3] Borja Requena,[3] Yusef Ahsini,[4] Solomon Asghar,[5] Jaeyong Bae,[6] Francisco J. Barrantes,[7] Steen W. B. Bender,[8,9] Clément Cabriel,[10] J. Alberto Conejero,[4] Marc Escoto,[11] Xiaochen Feng,[12] Rasched Haidari,[13,14] Nikos S. Hatzakis,[8,9] Zihan Huang,[15] Ignacio Izeddin,[10] Hawoong Jeong,[6,16] Yuan Jiang,[12] Jacob Kæstel-Hansen,[8,9] Judith Miné-Hattab,[17] Ran Ni,[18] Junwoo Park,[17] Xiang Qu,[15] Lucas A. Saavedra,[7] Hao Sha,[12] Nataliya Sokolovska,[17] Yongbing Zhang,[12] Giorgio Volpe,[5] Maciej Lewenstein,[3,19] Ralf Metzler,[20,21] Diego Krapf,[22] Giovanni Volpe,[2,23,†] and Carlo Manzo[24,25,‡]

[1]*Institute for Theoretical Physics, University of Innsbruck, Technikerstr. 21a, A-6020 Innsbruck, Austria*
[2]*Department of Physics, University of Gothenburg, Origovägen 6B, SE-41296 Gothenburg, Sweden*
[3]*ICFO – Institut de Ciències Fotòniques, The Barcelona Institute of Science and Technology, Av. Carl Friedrich Gauss 3, 08860 Castelldefels (Barcelona), Spain*
[4]*Instituto Universitario de Matemática Pura y Aplicada, Universitat Politècnica de València, València, Spain*
[5]*Department of Chemistry, University College London, 20 Gordon Street, WC1H 0AJ London, United Kingdom*
[6]*Department of Physics, Korea Advanced Institute of Science and Technology, Daejeon 34141, Korea*
[7]*Molecular Neurobiology Division, BIOMED UCA-CONICET, Buenos Aires C1107AAZ, Argentina*
[8]*Department of Chemistry, University of Copenhagen, Copenhagen, Denmark*
[9]*Novo Nordisk Center for Optimised Oligo Escape and Control of Disease, University of Copenhagen, Copenhagen, Denmark*
[10]*Institut Langevin, ESPCI Paris, Université PSL, CNRS, 75005 Paris, France*
[11]*Centro de Investigación en Gestión e Ingeniería de Producción, Universitat Politècnica de València, València, Spain*
[12]*School of Computer Science and Technology, Harbin Institute of Technology (Shenzhen), Shenzhen, China*
[13]*Gene Machines Group, Clarendon Laboratory, Department of Physics, University of Oxford, Oxford, UK*
[14]*Kavli Institute of Nanoscience Discovery, University of Oxford, Dorothy Crowfoot Hodgkin Building, Oxford, UK*
[15]*School of Physics and Electronics, Hunan University, Changsha 410082, China*
[16]*Center of Complex Systems, Korea Advanced Institute of Science and Technology, Daejeon 34141, Korea*
[17]*Sorbonne Université, CNRS, Laboratory of Computational Quantitative and Synthetic Biology (CQSB), F-75005 Paris, France*
[18]*School of Chemistry, Chemical Engineering and Biotechnology, Nanyang Technological University, 62 Nanyang Drive, 637459, Singapore*
[19]*ICREA, Pg. Lluís Companys 23, 08010 Barcelona, Spain*
[20]*Institute for Physics & Astronomy, University of Potsdam, Karl-Liebknecht-Str 24/25, D-14476 Potsdam-Golm, Germany*
[21]*Asia Pacific Centre for Theoretical Physics, Pohang 37673, Republic of Korea*
[22]*Department of Electrical and Computer Engineering and School of Biomedical Engineering, Colorado State University, Fort Collins, Colorado 80523, USA*
[23]*Science for Life Laboratory, Department of Physics, University of Gothenburg, Origovägen 6B, SE-41296 Gothenburg, Sweden*
[24]*Facultat de Ciències, Tecnologia i Enginyeries, Universitat de Vic – Universitat Central de Catalunya (UVic-UCC), C. de la Laura,13, 08500 Vic, Spain*
[25]*Institut de Recerca i Innovació en Ciències de la Vida i de la Salut a la Catalunya Central (IRIS-CC), 08500 Vic, Spain*

[*] gorka.munoz-gil@uibk.ac.at
[†] giovanni.volpe@physics.gu.se
[‡] carlo.manzo@uvic.cat

Method	Output level	Type of Approach	Diffusion parameters	Diffusion classes
Hidden Markov method with measurement noise propagation [1, 2]	Single trajectory	Stats	D	confined/Brownian
Hidden Markov method and simulations [3]	Single trajectory	Stats	D, α	
Supervised trajectory segmentation algorithm with support vector machine [4]	Single trajectory	ML		confined/drift/directed
Deep learning and moment scaling spectrum [5]	Single trajectory	ML + Stats	D	immobile/Brownian (fast/slow)
Piecewise linear approximation [6]	Single trajectory	Stats	velocity	
Detection of velocity and diffusion coefficient[7, 8]	Single trajectory	Stats	D, velocity	Brownian/directed
Back-propagation neural network [9]	Single trajectory	ML		confined/directed
Divide-and-conquer moment scaling spectrum [10]	Single trajectory	Stats	D, α, confinement radius	immobile/confined/Brownian/directed
Local convex hull [11]	Single trajectory	Stats	D,α	
Recurrence analysis [12]	Single trajectory	Stats	D,α	
Gamma mixture and a hidden Markov method [13]	Single trajectory	Stats + ML	D	
Superstatistical framework [14]	Single trajectory	Stats	activity, persistence	
Probability distribution of square displacements [15]	Ensemble	Stats	D, α	immobile/confined/Brownian/directed
Probability of transient confinement zones [16]	Single trajectory	Stats		confined/Brownian
Probability of jumps and transient confinement zones [17]	Single trajectory	Stats		confined/Brownian + jumps
Packing coefficient [18]	Single trajectory	Stats		immobile/confined/Brownian
Nonparametric Bayesian inference [19]	Ensemble	Stats + ML	D	
Displacement distribution and autocorrelation of squared displacements [20]	Ensemble	Stats	D	
Hierarchical Dirichlet process modeling [21]	Single trajectory	Stats	force	
Sliding window of time-averaged MSD [22]	Single trajectory	Stats	D,α	
Random forest [23]	Single trajectory	Stats		confined/Brownian/directed/anomalous
Gyration quantification and Bayesian statistics [24]	Single trajectory	Stats	D	fast/slow
Variational Bayesian treatment of hidden Markov method [25]	Ensemble	Stats	D	
Bayesian model selection of hidden Markov method [26]	Single trajectory	Stats	D	Brownian/directed
Measurement of anomalous diffusion using recurrent neural networks [27]	Single trajectory	ML	α	
Pointwise diffusion properties with transformers [28]	Single trajectory	ML	D and α	
Graph-neural network with attention [29]	Single trajectory & Ensemble	ML	D, α	
Recurrent neural network-based autoencoders [30]	Single trajectory	ML	D, α	
Bayesian deep learning [31]	Single trajectory	ML	α	
Semantic segmentation with convolutional networks [32]	Single trajectory	ML	α	

Supplementary Table 1. **Methods for the analysis of heterogeneous diffusion.** Methods for the analysis of heterogeneous diffusion are classified based on the level of output provided (single-trajectory methods vs. ensemble methods) and on the type of approach (machine learning (ML), classical statistics (Stats), or a combination of the two). We also indicate the diffusion parameters that each method estimates and the diffusion classes that each method uses to classify the trajectories. Only methods accounting for switches of diffusive behavior within the same trajectory are included.

		Ranking				Method			Members
		Video		Trajectory		Video	Trajectory		
	Team Name	ST	Ens	ST	Ens		ST	Ens	
A	AIntgonnawork	-	-	14	-	-	Sliding window	-	Thomas Martynec, Sarah A.M. Loos
B	EMetBrown	-	-	9	9	-	Point-wise inference	GMM-like	Maxime Lavaud, Juliette Lacherez, Yosef Shokeeb, Yacine Amarouchene, Thomas Salez
C	HNU	-	-	3	-	-	Point-wise inference	-	Zihan Huang, Xiang Qu
D	Unfriendly AI	-	-	7	8	-	Sliding window	GMM	Roman Lavrynenko, Lyudmyla Kirichenko, Sophia Lavrynenko
E	SPT-HIT	3	1	2	5	Trackmate [33]	Point-wise inference	GMM	Xiaochen Feng, Yuan Jiang, Hao Sha, Yongbing Zhang
F	BIOMED-UCA	-	2	10	10	DeepTrack [34] + trackpy [35]	Point-wise inference	GMM	Lucas A. Saavedra, Francisco J. Barrantes
G	KNU-ON	-	-	12	-	-	Point-wise inference	-	Taegeun Song, Seunghee Han, Jae-Hyung Jeong, Jihye Kim
H	Nanoninjas	-	-	11	3	-	Point-wise inference	Clustering	Gabriel Fernández-Fernández, Borja Requena
I	UCL SAM	-	-	1	1	-	Point-wise inference	GMM	Solomon Asghar, Ran Ni, Giorgio Volpe
J	far_naz	-	-	18	-	-	N/A	-	Farzaneh Nazari, Mohammad Mehdi Nazari
K	ICSO-UPV	2	2	15	6	Crocker-Grier[36]	Point-wise inference	Clustering	Yusef Ahsini, Marc Escoto, J. Alberto Conejero
L	HSC AI	-	-	13	7	-	CP analysis	Clustering	Janusz Szwabiński, Jakub Malinowski, Marcin Kostrzewa, Michał Balcerek, Weronika Tomczuk
M	DeepSPT	-	-	17	2	-	Point-wise inference	GMM	Jacob Kæstel-Hansen, Nikos S. Hatzakis, Steen W. B. Bender
N	KCL	-	-	8	11	-	CP analysis	Clustering	Alvaro Lanza, Stefano Bo
O	CSSPL	-	-	5	4	-	CP analysis	GMM	Jaeyong Bae
P	D.AnDi	-	-	16	-	-	Point-wise inference	-	Raffaele Pastore, Francesco Rusciano, Maurizio De Micco, Pier Luca Maffettone, Francesco Greco
Q	SU-FIONA	1	-	6	-	Freetrace (unpublished)	Sliding window	-	Junwoo Park, Nataliya Sokolovska, Ignacio Izeddin, Clément Cabriel, Judith Miné-Hattab
R	M3	-	-	4	-	-	Point-wise inference	-	Rasched Haidari

Supplementary Table 2. **Summary of the participating teams and submitted methods.** The "Ranking" columns report the position of the team in the final leaderboard of each Track and Task. ST refers to the Single-trajectory Task and Ens to the Ensemble Task. The top 5 teams are highlighted in gray. The "Method" column includes a high-level description of the methods used for each Track and Task. The "Video" column only summarizes the method used for extracting the trajectories, since all the teams participating in the Video Track performed further analysis with the same approach used for the Trajectory Track, which is described in column "Trajectory". For the Single-trajectory Task (subcolumn "ST"), we could identify three approaches: "CP analysis", i.e., methods predicting the position of the changepoints from the raw data; "Point-wise inference", i.e., methods that predict the diffusive properties at each frame (see [28]); "Sliding window", i.e., method that predict either changepoints or diffusive properties by averaging or filtering over a sliding window. For the Ensemble Task (subcolumn "Ens"), all teams obtained an estimation of the parameters by pooling predictions obtained at the single-trajectory level, either using a Gaussian mixture model ("GMM") or a clustering algorithm ("Clustering").

Experiment	Model	Numb. of states	State	K ($\text{pixel}^2/\text{frame}^\alpha$)		α distrib.		Diffusion class	Model-specific parameters
				μ	σ^2	μ	σ^2		
1	MSM	3	1	0.15	0.01	1	0.0001	2	$M = \begin{pmatrix} 0.946 & 0.024 & 0.03 \\ 0.016 & 0.92 & 0.064 \\ 0.037 & 0.14 & 0.823 \end{pmatrix}$
			2	0.33	0.001	1	0.01	2	
			3	0.95	0.01	1	0.01	2	
2	DIM	2	1	0.28	0.001	1	0.1	2	$r = 0.5,\ N = 750,\ P_\text{b} = 1,\ P_\text{u} = 0.1$
			2	0.0035	0.0001	1.1	0.01	2	
3	QTM	2	1	1	0.1	1	0.005	2	$r_\text{t} = 0.4,\ N_\text{t} = 500,\ P_\text{b} = 0.7,\ P_\text{u} = 1/60$
			2	0	0	0	0	0	
4	TCM	2	1	1	0.1	1	0.005	2	$r_\text{c} = 2,\ N_\text{c} = 150,\ T = 0.1$
			2	0.01	0.001	0.2	0.001	1	
5	DIM	2	1	1	0.1	1	0.005	2	$r = 0.35,\ N = 500,\ P_\text{b} = 1,\ P_\text{u} = 1/60$
			2	0.01	0.001	0.2	0.001	2	
6	DIM	2	1	0.1	0.1	0.7	0.1	2	$r = 1,\ N = 100,\ P_\text{b} = 1,\ P_\text{u} = 0.05$
			2	1	0.01	1.2	0.01	2	
7	MSM	2	1	0.1	0.1	0.7	0.1	2	$M = \begin{pmatrix} 0.98\overline{3} & 0.01\overline{6} \\ 0.05 & 0.95 \end{pmatrix}$
			2	1	0.01	1.2	0.01	2	
8	SSM	1	1	1	100	1	10	2 / 3	-
9	QTM	2	1	1	0.01	1.99	0.01	2 / 3	$r_\text{t} = 1.5,\ N_\text{t} = 1000,\ P_\text{b} = 0.7,\ P_\text{u} = 1/5$
			2	0	0	0	0	0	

Supplementary Table 3. **Parameters of the Challenge dataset.** Specifications of the dataset of the last phase of the 2nd Anomalous Diffusion Challenge: *single-state model* (SSM); *multi-state model* (MSM); *dimerization model* (DIM); *transient-confinement model* (TCM); *quenched-trap model* (QTM). The diffusion class correspond to 0 = immobile, 1 = confined, 2 = free (unconstrained), 3 = directed. The parameters specific to each theoretical model are: for MSM, the transition matrix M; for QTM, the trap radius r_t, the number of traps N_t, the probability of trapping P_b and untrapping P_u; for DIM, the interactions radius r, the number of particles N, the probability of binding P_b and unbinding P_u; for TCM, the compartment radius r_c, the number of compartments N_c, and the boundary transmittance T. Simulations are provided in generalized units (i.e., pixels and frames) that can be rescaled to meaningful temporal and spatial scales. See Methods for further details. For all experiments, we simulated $N = 100$ particles (Experiment 7 has $N = 80$) in a box of size $L = 230.4$ pixels with a FOV size $L_\text{FOV} = 128$ pixel, and a maximum trajectory length of 200 frames. For Video Track, movies were rendered using a $\text{FWHM}_\text{PSF} = 2.1$ pixels and a SNR = 7.1. For the Trajectory Track, trajectories were corrupted with Gaussian localization noise with $\sigma_N = 0.12$ pixels.

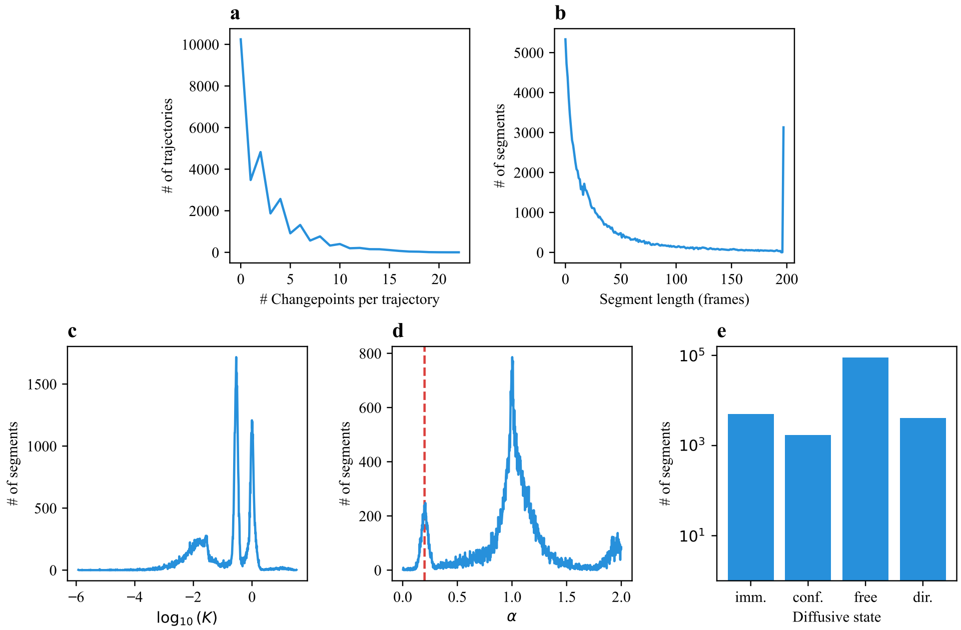

Supplementary Figure 1. **Distribution of specific features over the whole Challenge dataset.** **a** Number of trajectories as a function of the number of changepoints for trajectory. **b** Number of segments as a function of their duration. **c** Number of segments as a function of their α. **d** Number of segments as a function of their K. **e** Number of segments as a function of their diffusive state.

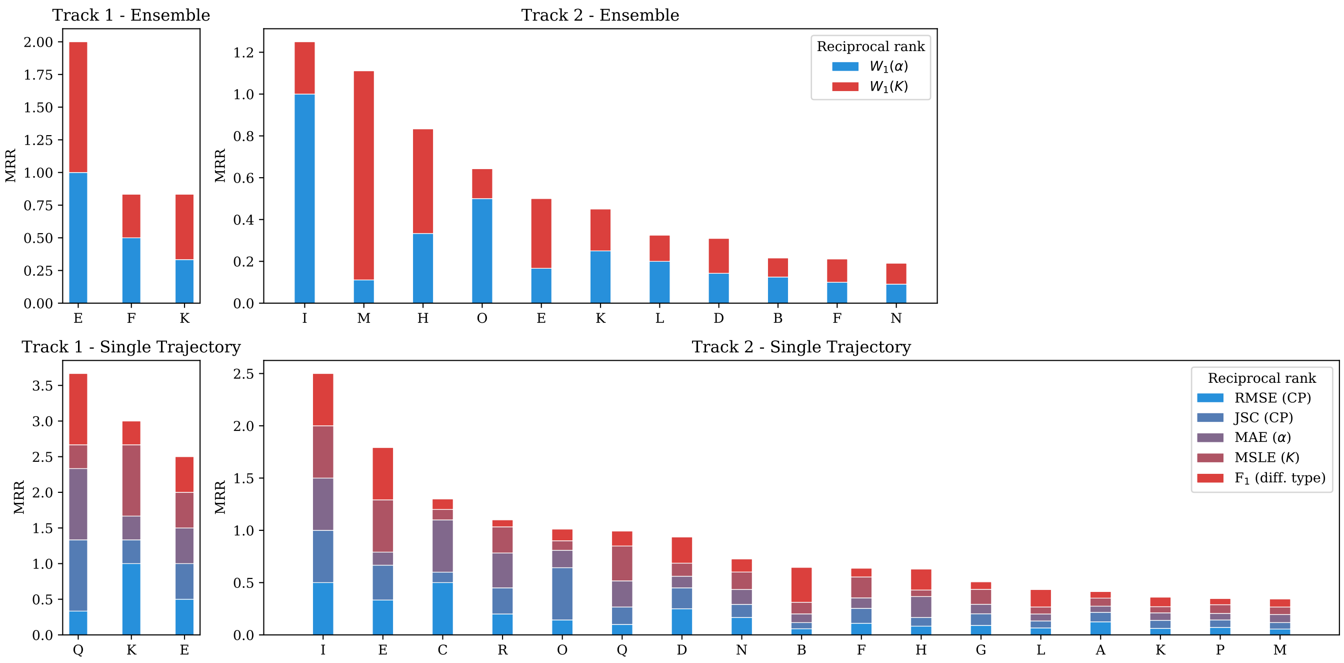

Supplementary Figure 2. **Mean reciprocal rank (MRR) of all the methods for each Track and Task.** The colors represent the relative contributions of the metrics of each subtask to the overall MRR.

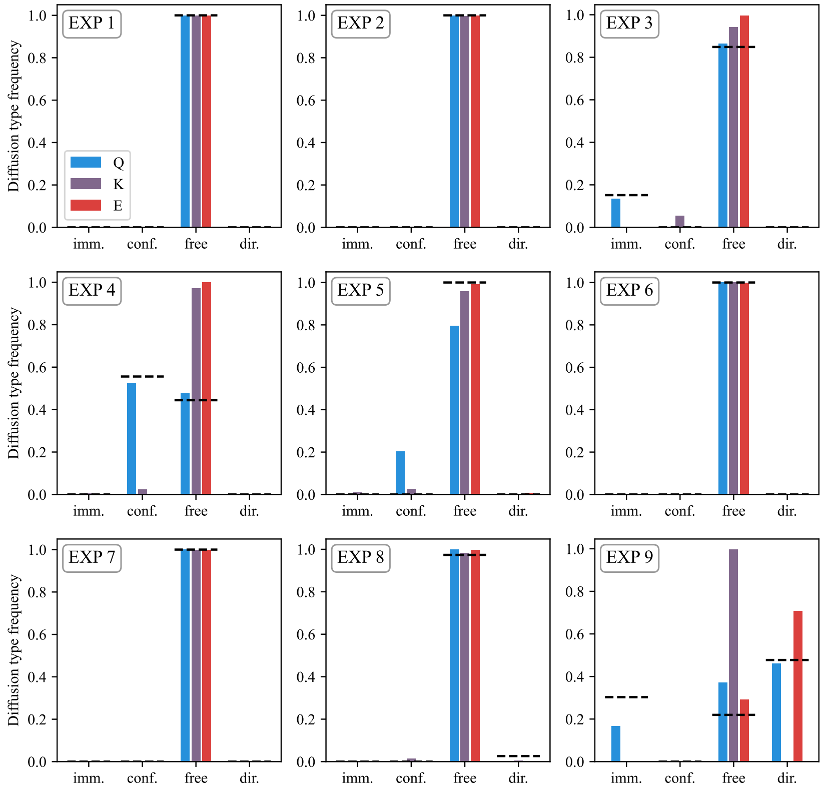

Supplementary Figure 3. **Detailed results of the Single-trajectory Task of the Video Track.** Predictions for the frequency of time spent with a given diffusion type for each experiment for the Single-trajectory Task of Video Track. Only the results of the top 5 teams are shown. Dashed black lines represent the ground truth.

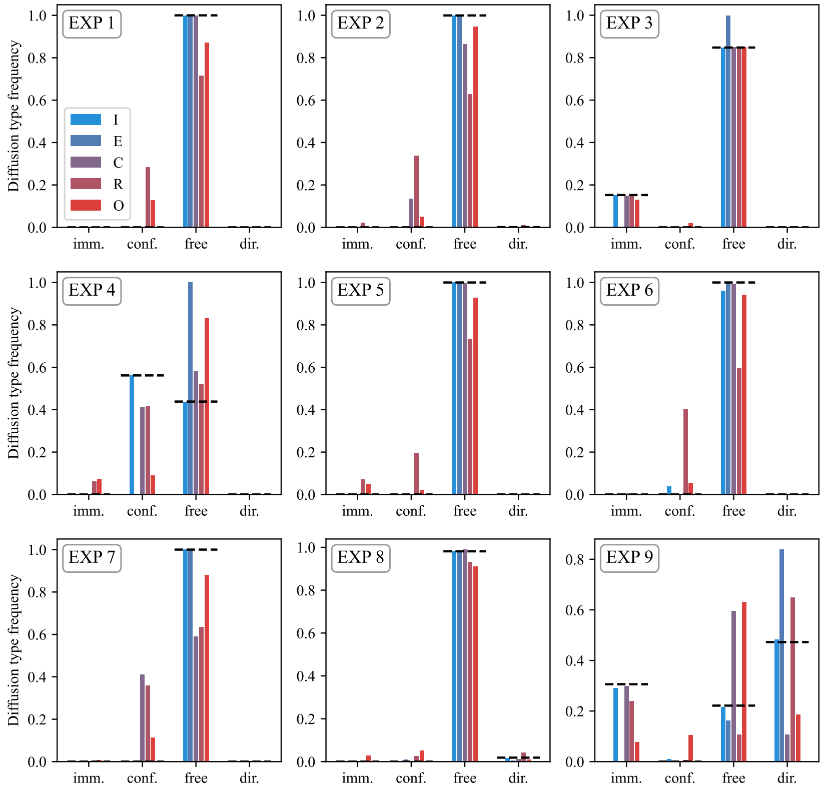

Supplementary Figure 4. **Detailed results of the Single-trajectory Task of the Trajectory Track.** Predictions for the frequency of time spent with a given diffusion type for each experiment for the Single-trajectory Task of the Trajectory Track. Only the results of the top 5 teams are shown. Dashed black lines represent the ground truth. Abbreviations stand for: imm.: immobile, conf.: confined and dir.: directed

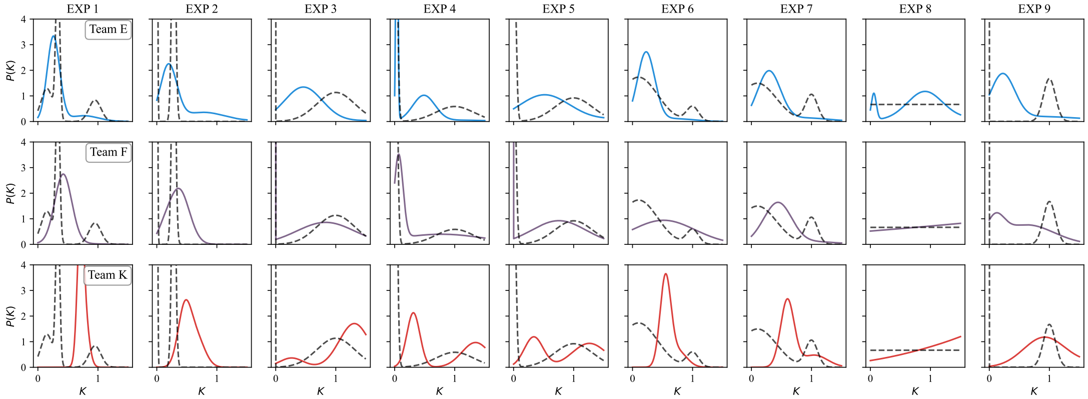

Supplementary Figure 5. **Detailed results of the Ensemble Task for the Video Track.** Predictions for the distributions of K for each experiment of the Ensemble Task for the Video Track. Dashed black lines represent the ground truth.

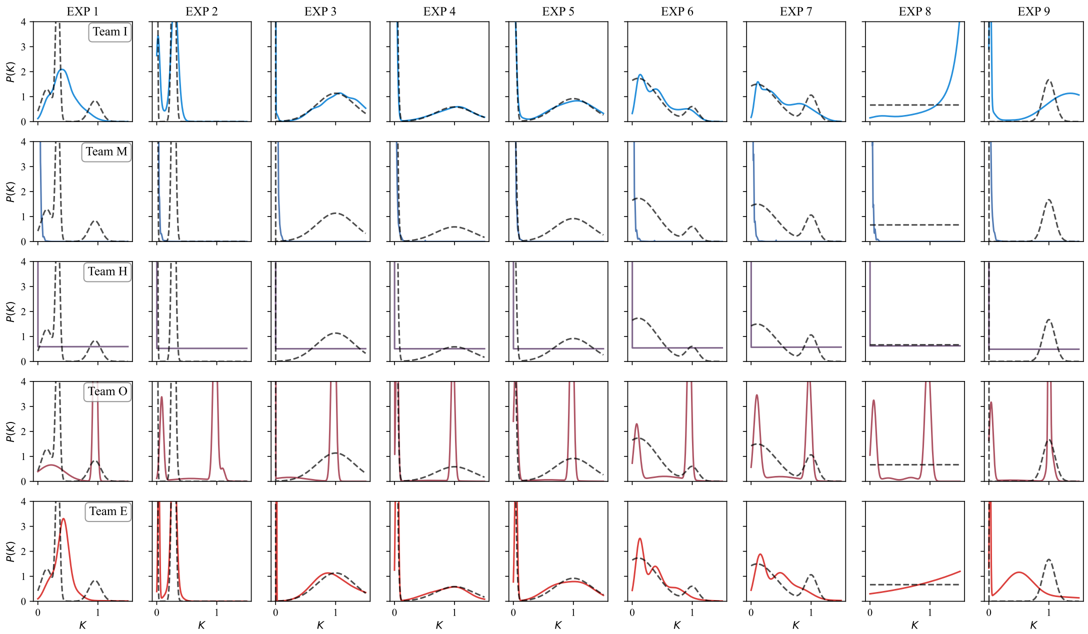

Supplementary Figure 6. **Detailed results of the Ensemble Task for the Trajectory Track.** Predictions for the distributions of K for each experiment of the Ensemble Task for the Trajectory Track. Only results of the top 5 teams are shown. Dashed black lines represent the ground truth.

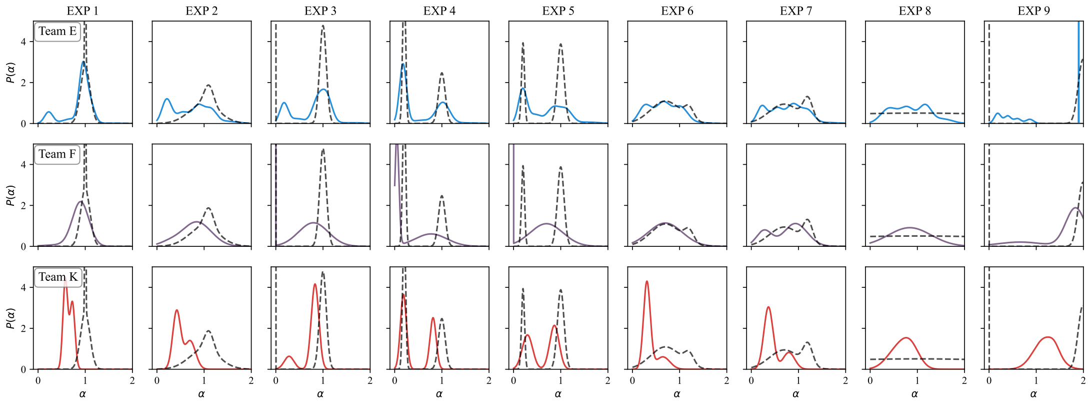

Supplementary Figure 7. **Detailed results of the Ensemble Task for the Video Track.** Predictions for the distributions of α for each experiment of the Ensemble Task for the Video Track. Dashed black lines represent the ground truth.

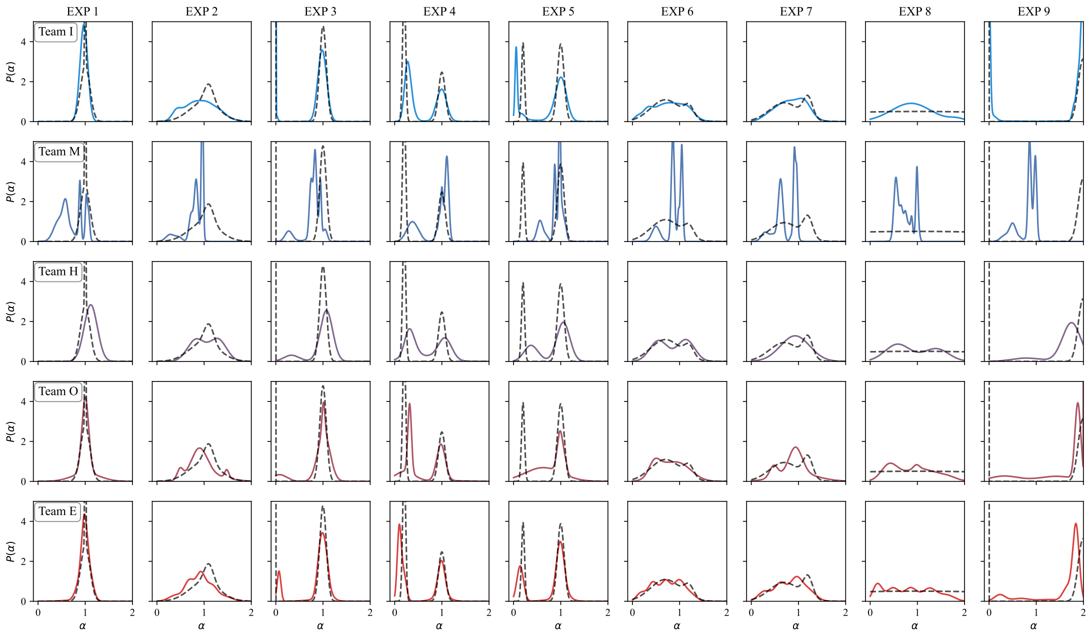

Supplementary Figure 8. **Detailed results of the Ensemble Task for the Trajectory Track.** Predictions for the distributions of α for each experiment of the Ensemble Task for the Trajectory Track. Only the results of the top 5 teams are shown. Dashed black lines represent the ground truth.

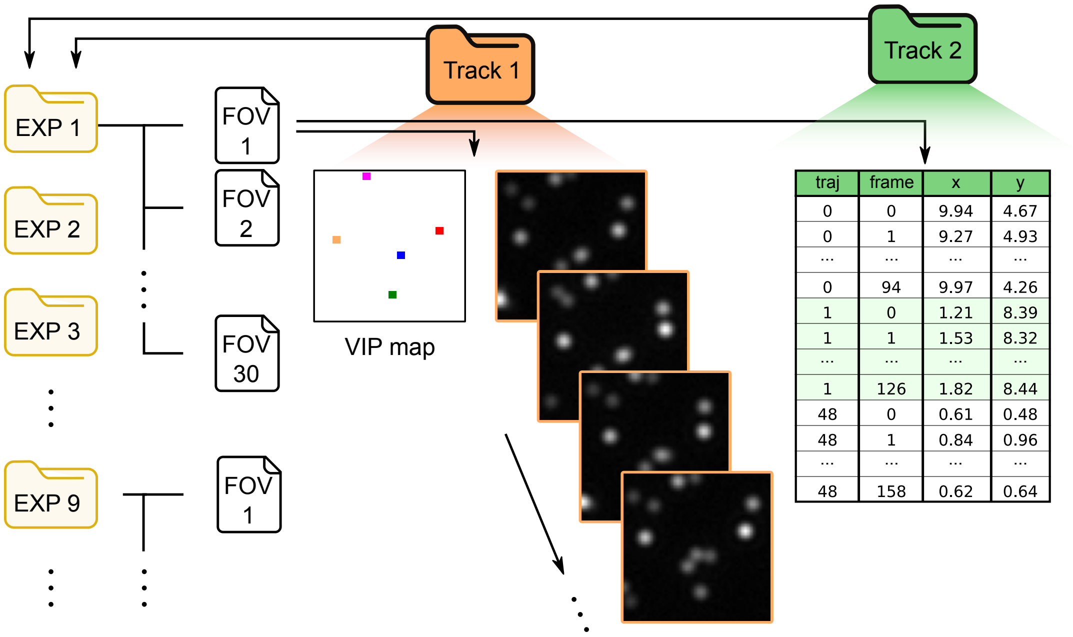

Supplementary Figure 9. **Structure of the dataset.** Datasets for each track are contained in the respective directories, namely, `Video Track` and `Trajectory Track`. These include a folder for each experiment labeled with a sequential number (`EXP_[exp number]`). Each experiment folder contains a list of 30 files labeled with a sequential number (`FOV_[fov number]`) associated with different non-overlapping FOVs. For the Video Track, the files consist of 200-frame videos and an additional map corresponding to the segmentation of the very important particles (VIP) at the first frame. For the Trajectory Track, the files contain a table whose columns correspond to the trajectory index, time step, x-coordinate, and y-coordinate.

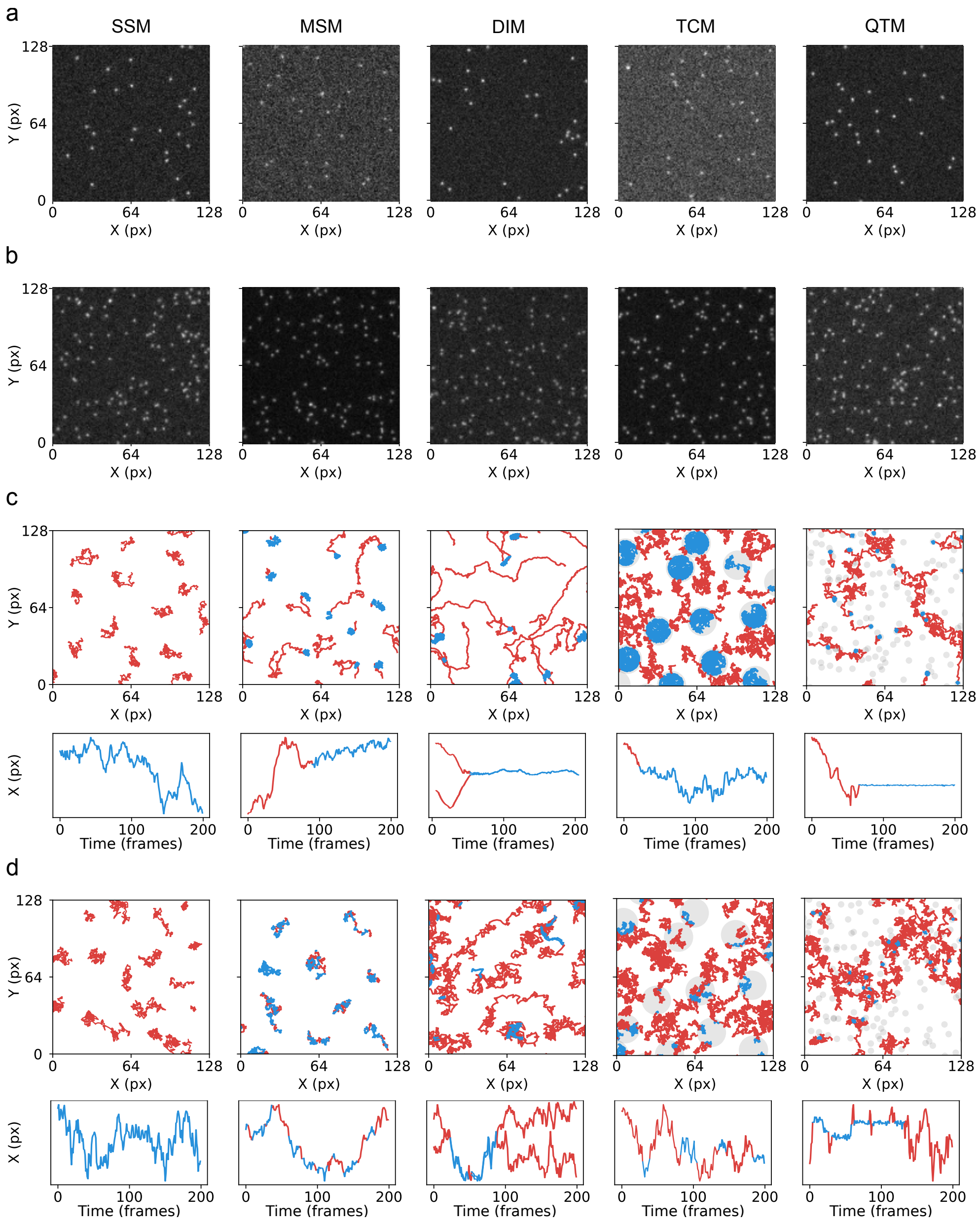

Supplementary Figure 10. **Examples of Challenge datasets.** **a, b**, Frames of exemplary videos (FOVs) provided for the Video Track corresponding to the different diffusion models and reproducing low (**a**) and high number density (**b**). **c, d**, The upper rows show plots of the trajectories for exemplary FOVs provided for the Trajectory Track corresponding to the different diffusion models and different sets of parameters exhibiting evident (**c**) or subtle changes (**d**) of diffusion, i.e., data in **c** reproduce conditions where diffusion changes are easier to identify as compared to **d**. For example, for the SSM, all trajectories in **c** have the same generalized diffusion coefficient, whereas in **d** they have a broad distribution of K. For the MSM, trajectories in **c** undergo large changes of diffusivity and show long dwell times in each state, as compared to **d**. For the DIM, trajectories in **c** undergo large changes of anomalous diffusion exponent and show long dwell times in each state, as compared to **d**. For the TCM, in **c**, trajectories cannot leave the confinement zone once they get inside, whereas in **d** the compartments have a finite transmittance. For the QTM, the dwell times in the traps are much longer in **c** than in **d**. The lower rows show time traces of the x-coordinate of representative trajectories. The different colors indicate different diffusive states (imm.: immobile, conf.: confined, free: free, dir.: directed).

OVERVIEW OF TEAMS AND METHODS

In this section, we provide a brief overview of the teams and the methods they employed to participate in the Challenge, as written by the team members. Whenever available, references to the published versions of these methods are included.

Team C: *HNU*	
Contact:	Zihan Huang, Xiang Qu<br>School of Physics and Electronics, Hunan University, Changsha 410082, China
Reference:	[32, 37]
Method:	WADNet + U-AnDi
Platform:	Python + PyTorch
Open-access:	https://huggingface.co/huangzih/AnDiChallenge2
Description:	Our method for the single trajectory task in track 2 combines WADNet [37] (for classification and regression) and U-AnDi [32] (for segmentation). The steps are as follows:<br>1. Initial classification with WADNet: WADNet classifies each experiment as SSM, MSM, QTM, or TCM. For DIM, visual inspection suffices without WADNet.<br>2. Traing WADNet and U-AnDi: For each classified experiment, independent models are trained using both WADNet and U-AnDi: a) Classification model (WADNet): Identifies if there are changepoints within the trajectory. b) Regression model (WADNet): Estimates the diffusion exponent (alpha) and the diffusion coefficient (K) directly. c) Segmentation model based on alpha (U-AnDi). d) Segmentation model based on K (U-AnDi).<br>3. Changepoint detection and parameter estimation: For SSM, MSM, QTM, or TCM, the following steps are employed: a) Determine the presence of changepoints using the classification model (2.a). b) If no changepoints are present, directly output alpha and K using the regression model (2.b). c) If changepoints exist, derive alpha and K for each point along the trajectory using models (2.c) and (2.d). d) Apply the post-processing technique in Ref. [32] to determine changepoints based on the variations in alpha. e) Segment the trajectory based on these changepoints, and calculate the average values of alpha and K from step (3.c) for each segment.<br>4. Special case for DIM: a) Changepoints are identified by examining the distance between points of two trajectories in the same frame. b) Trajectories are then segmented based on these changepoints. Alpha and K of each segment (length ≥ 20) are estimated using the regression model (2.b). c) For very short segments (length < 20), we use the average values of alpha and K from the entire experiment as their predictive values.

Team E: *SPT-HIT*	
Members:	Xiaochen Feng, Yuan Jiang, Hao Sha, Yongbing Zhang<br>School of Computer Science and Technology, Harbin Institute of Technology (Shenzhen), Shenzhen, China.
Reference:	-
Method:	U-LSTMFormer
Platform:	Python
Open-access:	https://github.com/AlphaYuan/SPT-HIT-code
Description:	We present a novel pipeline, U-LSTMFormer, for accurate single-molecule motion analysis in Video (Ensemble and Single-trajectory tasks) and Trajectory (Ensemble and Single-trajectory tasks) tracks. The pipeline consists of three stages: video trajectory linking, trajectory statement prediction, and ensemble analysis.<br>*Video Trajectory Linking* Using TrackMate [33], an ImageJ plugin, we generate molecule trajectories by linking particles in videos for all Video tasks. The LAP tracker is applied with optimized parameters to preferentially link trajectories.<br>*Trajectory statement Prediction* We developed a U-shaped network combining LSTM [38] and Transformer [39] architectures to predict the diffusion coefficient (K) and anomalous exponent (α) at each point in the trajectory for all tasks. The network processes differences in trajectory coordinates at various time lags, passing them through down-sampling layers (1D convolution and average pooling) for multi-timescale feature extraction. The down-sampling layers reduce the input length while increasing the channel depth. The output from the lowest down-sampling layer is processed by a Transformer Encoder, while the outputs from earlier layers are handled by a Gated-LSTM encoder for temporal feature extraction. These outputs are then combined through up-sampling and 1D convolution, producing a feature vector of the same length as the original trajectory. Finally, two linear heads output the values of K and α. Changepoint detection is performed on K and α using the kernelCPD method [40] from the ruptures library. Training uses datasets $\mathcal{D} = \{(\mathbf{x}_i, y_i) \| i \in [1, 5e^5]\}$ sampled from andi-datasets [**?**] with different trajectory length $L \in [10, 200]$ and a designed training objective $\mathbb{E}_{(\mathbf{x},y)\sim\mathcal{D}}[\mathbf{M} \cdot \mathcal{L}_{MAE}(f(\mathbf{x}), y)]$ where $\mathbf{M}$ is a fixed point-to-point weight matrix determined by ground-truth segment lengths, change point positions, and state transitions.<br>*Ensemble Analysis* To analyze K and α, which follow distinct Gaussian distributions, we apply a Gaussian Mixture Model (GMM) [41] and Bayesian Information Criterion (BIC) [42] for optimal component selection. The data for K and α are then allocated to Gaussian components, and their distributions over time segments are analyzed to derive final results for all Ensemble tasks.

Team F: *Molecular Neurobiology, BIOMED UCA-CONICET*	
Members:	Francisco J. Barrantes, Lucas A. Saavedra<br>Laboratory of Molecular Neurobiology, BIOMED UCA-CONICET, Buenos Aires C1107AAZ, Argentina
Reference:	-
Method:	tWadTCN
Platform:	Python
Open-access:	https://github.com/lucasSaavedra123/wadtcn
Description:	*Trajectory Track - Single-trajectory Task* tWadTCN is a method comprising three neural networks (NNs) to predict the diffusion coefficient, alpha exponent, and state at each point along a trajectory. NNs are composed of three pre-established components for feature extraction: a WadNet encoder to extract long-time correlated features from the trajectories, in a manner similar to that used in [37], but having a decreased dilation depth; another feature extractor employing multi-channel convolutions (as in [43] and [44]) and skip connections to extract additional features that has proved to be a general feature extractor for NNs dedicated to trajectory diffusion analysis; and a simplified transformer module (introduced by [28]). To detect change point detections, we used AutoStepFinder, which was applied to each single-level predictions returned by the NNs and subsequently combined [45]. The architecture combines and significantly simplifies components from various studies for enhanced inference and training efficiency. The neural network dedicated to predicting diffusion coefficients transforms trajectories into their corresponding displacements along the 'x' and 'y' axes before making predictions. In contrast, the other NNs receive the raw trajectories without transformations. All training trajectories were simulated using the andi-datasets software package. NNs that predict the diffusion coefficient and alpha exponent were trained exclusively on multi-state trajectories with varying diffusion coefficients and alpha values within the specified range in the challenge. The NN predicting the state at each point along trajectories was trained across a wide range of simulation parameters, encompassing single-state, multi-state, confinement, and immobilization scenarios. Due to time constraints, dimerization trajectories were excluded from the training set.<br>*Video Track - Single-trajectory Task* For videos, trajectories were extracted with a UNet-based particle localizer [34] and particle linking algorithm implemented in trackpy [35]. These trajectories were further analyzed as the ones in the Trajectory Track.<br>*Video/Trajectory Track - Ensemble Task* For ensemble tasks, we aggregated all individual point-wise predictions from video or trajectory track and visually inspected their distributions using histograms. Subsequently, a simple Gaussian mixture model for each experiment was applied to identify the mean and standard deviation within these distributions.

Team H: *Nanoninjas*	
Members:	Gabriel Fernández-Fernández, Borja Requena<br>ICFO – Institut de Ciències Fotòniques, The Barcelona Institute of Science and Technology, Av. Carl Friedrich Gauss 3, 08860 Castelldefels (Barcelona), Spain
Reference:	[28]
Method:	KISTEP
Platform:	Python
Open-access:	https://github.com/GabrielFernandezFernandez/kistep
Description:	KISTEP combines a single-point single-trajectory characterization method, STEP [28], with a Gaussian kernel change-point detection [46] for trajectory segmentation, and k-means [47] to cluster those segments according to their properties.<br>*Trajectory Track - Single-trajectory Task* We train a STEP machine on simulated trajectories for each of the relevant trajectory properties of the challenge, i.e., the anomalous exponent (STEP_α), the diffusion coefficient ($\text{STEP}_{\log_{10} K}$), and the state ($\text{STEP}_{\text{state}}$). We enhance the original STEP implementation by engineering a rotationally invariant representation of the trajectories in polar coordinates, consisting of the displacement module and the angle between successive displacements. These features are the inputs for STEP_α, and we take the logarithm in base 10 of the displacements' module for $\text{STEP}_{\log_{10} K}$. $\text{STEP}_{\text{state}}$ follows the original STEP, taking directly the Cartesian coordinates in pixel units as input.<br>Following the proposal from [28], we segment STEP's point-wise predictions with a Gaussian kernel change-point detector [46]. We perform the segmentation taking into account all predicted properties (α, K and state), and further refine it by merging successive segments with an absolute difference lower than 0.1 in α and $\log_{10}(K)$.<br>*Trajectory Track - Ensemble Task* To predict the ensemble means and standard deviations, we cluster the previously segmented STEP predictions with the k-means [47] algorithm. We determine the number of clusters by performing a knee search combined with visual inspection of the clustering weighted by residence time. Additionally, we discard clusters representing less than 2% of segments.<br>Further methods for video analysis were prototyped, but were not developed enough to yield satisfactory predictions. All the methods can be seen in the GitHub repository, with training and inference examples.

Team I: *UCL SAM*	
Members:	Giorgio Volpe[1], Solomon Asghar[1], Ran Ni[2]<br>[1] Department of Chemistry, University College London, 20 Gordon Street, WC1H 0AJ London, United Kingdom<br>[2] School of Chemistry, Chemical Engineering and Biotechnology, Nanyang Technological University, 62 Nanyang Drive, 637459, Singapore
Reference:	[48]
Method:	U-AnD-ME (UNet 3+ for Anomalous Diffusion analysis enhanced with Mixture Estimates)
Platform:	Python + TensorFlow
Open-access:	https://doi.org/10.5281/zenodo.14360592
Description:	*Trajectory Track* Our method uses a UNet3+-inspired neural network [49] to make per-timestep predictions for trajectories [37]. Per-timestep predictions are processed into segment-level predictions based on change points. Additionally, combining predictions from all trajectories across an experiment enables phenomenological model prediction, and generation of a Gaussian mixture model (GMM) quantifying ensemble properties. To further increase prediction accuracy of single trajectories, experiment-specific networks are created, each trained on trajectories reflecting their experiment, i.e. of the predicted model with properties following the GMM. Training trajectories are generated using the andi-datasets package. Initially, trajectories corresponding to every model are generated, with parameters sampled from wide ranges. For experiment-specific networks, only trajectories from models deemed to be likely are generated, with α and K sampled following the experiment's GMM. We process each trajectory into a training sample by differencing along the time axis and padding to a length of 224 timesteps. Samples are padded with zeros and corresponding labels are padded with zeros barring diffusion type, which we assign as "transient-confinement model", effectively treating the FOV boundary as an immobilizing trap. Once 50,000 samples are generated, training proceeds until validation loss stagnates for 3 epochs. Then, new samples are generated and another training iteration occurs. Training comes to a final stop when validation loss stagnates for 3 iterations. At each timestep, we output: a sigmoid for changepoint probability, a 5-way softmax for model prediction, and linear outputs for α, K, and diffusion type. After reversing padding, outputs are segmented according to their predicted change points. The values of α, K and diffusion type for each timestep across a segment are averaged to generate a singular prediction for that segment. An experiment's model is predicted by averaging over all softmax outputs across all its trajectories' timesteps. A GMM is generated using all timesteps' assigned α, K and diffusion type values.

Team K: *ICSO-UPV*	
Members:	J. Alberto Conejero[1], Yusef Ahsini[1], Marc Escoto[2]<br>[1] Instituto Universitario de Matemática Pura y Aplicada. Universitat Politècnica de València<br>[2] Centro de Investigación en Gestión e Ingeniería de Producción. Universitat Politècnica de València
Reference:	[50]
Method:	AnomaloUs-net
Platform:	Python
Open-access:	https://github.com/yusef320/AnDi2-ICSO_UPV
Description:	Our AnomaloUs-Net architecture comprises three main components: a particle tracking algorithm, an attention U-Net model, and a change-point method. With slight modifications we have used for both tracks and tasks.<br>(a) Initially, the video of the particle within the FOV is processed using the TrackPy library [35], which implements the Crocker-Grier algorithm for efficient particle detection and tracking [36]. Once the particles are detected, we link them across frames, creating continuous trajectories.<br>(b) The trajectories are then analyzed by three distinct Attention U-Net models to predict the α coefficient, the k coefficient, and the particle's state at each frame of the video. This architecture is an enhancement of the traditional U-Net [51] with attention mechanisms [52]. To enhance feature relevance, the network uses attention gates to selectively emphasize important features by integrating information from both the encoder and decoder paths [53].<br>(c) After generating the predictions, we will have three distinct time series for each particle in the field of view (FOV): α, k, and the state at each position. To detect changes in state, we will focus mainly on the α and k time series while using the state series to fine-tune the detection process. Specifically, particles in the ""trapped"" state exhibit α and k values close to zero, which helps us refine the change point detection method.<br>We analyze the particle's state at each time step. First, change points are identified by detecting when the trajectory shifts its predicted state. Later, we use the sliding window method implemented in the Ruptures Python library [54].

Team M: *DeepSPT*	
Members:	Nikos S. Hatzakis, Jacob Kæstel-Hansen, Steen W. B. Bender<br>Department of Chemistry & Nanoscience Center, University of Copenhagen, 1172 Copenhagen, Denmark
Reference:	[55]
Method:	DeepSPT
Platform:	Python
Open-access:	https://github.com/JKaestelHansen/AndiChallengeDeepSPT
Description:	*Trajectory Track - Single-trajectory Task* End-to-end UNET architecture with output nodes for alpha, D, and motion type per time point per track. Loss function being errors in predicting Alpha, D and motion type per timepoint. Alpha and D change points detected by absolute distance between neighbouring time points and a threshold. Trained on provided AnDi challenge simulation of tracks.<br>*Trajectory Track - Ensemble Task* For the ensemble prediction task, the alpha and D predictions per time point per track from the End-to-end UNET described above are for each experiment pooled. For each experiment, a Gaussian mixture models are fitted to the pooled alpha and D predictions individually defining the number of Gaussians to fit as the highest number of changepoints identified by the UNET for the tracks in the given experiment. The Gaussian mixture model fit returns the average, standard deviation, and the relative weights for each identified state.

Team O: *CSSPL*	
Members:	Hawoong Jeong[1,2], Jaeyong Bae[1]<br>[1] Department of Physics, Korea Advanced Institute of Science and Technology, Daejeon 34141, Korea<br>[2] Center of Complex Systems, Korea Advanced Institute of Science and Technology, Daejeon 34141, Korea
Reference:	[56]
Method:	Segment-based task-specific deep learning model framework
Platform:	Python
Open-access:	https://github.com/peardragon/AnDi2
Description:	*Trajectory Track - Single-trajectory Task* Our method utilizes five task-specific models, each derived from modified ResNet architectures tailored to two-dimensional trajectory data. These models are: the CP Existence Classifier, First CP Predictor, Diffusion Coefficient Predictor, Diffusion Exponent Predictor, and Diffusion State Classifier. The analysis proceeds iteratively as follows: The CP Existence Classifier initially determines whether change points (CPs) are present in the trajectory. Upon detecting a CP, the First CP Predictor estimates its position by predicting the step size from the trajectory's start. This segmentation divides the trajectory into a pre-CP segment (first segment) and a post-CP segment (residual trajectory). The Diffusion Coefficient, Exponent, and State Predictors detect the diffusion properties for the first segment. In the next step, the trajectory is modified by removing the first segment and replacing it with padded residuals, enabling the analysis to repeat. This iterative process continues until no CPs are detected. For trajectories without further CPs, the remaining segment bypasses the First CP Predictor and directly undergoes diffusion property analysis in a terminal phase.<br>*Trajectory Track - Ensemble Task* In the ensemble task, Gaussian Mixture Modeling (GMM) is applied. Segment-wise diffusion coefficients, exponents, and states obtained from the single-trajectory analysis are aggregated. The total number of properties obtained is calculated as the product of each trajectory's time steps and the number of trajectories in the experiment. For example, diffusion coefficients for individual segments are aggregated according to the number of time steps within each segment. GMM is performed on these aggregated properties, with the number of components corresponding to the unique state count. Bayesian Information Criterion (BIC) scores are used to select the best-fitting GMM. The resulting means and variances from the selected GMM represent the ensemble diffusion properties.

Team Q: *SU-FIONA*	
Members:	Junwoo Park[1], Nataliya Sokolovska[1], Ignacio Izeddin[2], Clément Cabriel[2], Judith Miné-Hattab[1]<br>[1] Sorbonne Université, CNRS, Laboratoire de Biologie Computationnelle et Quantitative, LCQB, F-75005 Paris, France<br>[2] Institut Langevin, ESPCI Paris, Université PSL, CNRS, 75005 Paris, France
Reference:	-
Method:	FreeTrace and BI-ADD
Platform:	Python and C
Open-access:	FreeTrace:https://github.com/JunwooParkSaribu/FreeTrace<br>BI-ADD:https://github.com/JunwooParkSaribu/BI_ADD
Description:	*Video Track - Single-trajectory Task* FreeTrace [57] infers the molecular trajectories in two folds. First is localizations of molecules from raw video. It detects positions of molecules at pixel-level by computing the difference between Gaussian PSF and Gaussian noise. From the computed difference, it fits 2D Gaussian distribution on the local maxima to calculate the positions of molecules at sub-pixel level. Second is reconnections of localized molecules. FreeTrace has two terms for the reconnection of molecules, jump distance and α(anomalous diffusion exponent). The approximated α supports the inference of reconnection if the length of trajectory becomes longer than 4. With α and jump distance, FreeTrace generates a multivariate Gaussian distribution with approximated α and jump distance to choose a particle at the next frame which has maximum likelihood among potential particles. The estimation of α is computed with ConvLSTM [58] from simulated noiseless fBm trajectories with andi-datasets.<br>*Trajectory Track - Single-trajectory Task* BI-ADD [59] segments a heterogeneous trajectory into sub-trajectories. BI-ADD converts a trajectory into a signal suggesting potential change-points with multiple sizes of sliding windows. From the sub-trajectories divided by change-points including false positives, it generates a global distribution of α and K. BI-ADD utilizes GMM(Gaussian mixture model) on the α and K distribution for the clustering. From the change-points, it compares the likelihoods of two consecutive sub-trajectories with clusters. If there is a significant change of likelihood or label between two consecutive sub-trajectories, BI-ADD merges the two consecutive sub-trajectories and considers the change-point as false positive, true positive otherwise. BI-ADD iterates this merging step until it checks every potential change-point. The estimations of K and α for the sub-trajectories are performed with simple neural network and ConvLSTM respectively, which are trained with noiseless fBm trajectories, generated with andi-datasets [**?**].

Team R: *M3*	
Members:	Rasched Haidari<br>Gene Machines Group, Clarendon Laboratory, Department of Physics, University of Oxford, Oxford, UK / Kavli Institute of Nanoscience Discovery, University of Oxford, Dorothy Crowfoot Hodgkin Building, Oxford, UK
Reference:	-
Method:	M3 - An LSTM approach for pointwise inference
Platform:	Python + PyTorch
Open-access:	https://github.com/raschedh/AnomalousDiffusion
Description:	M3 is a machine learning method using stacked bi-directional LSTMs (BiLSTMs). Specifically, the architecture consists of three BiLSTM cells with ReLU layers, skip connections, and layer normalizations, followed by a fully connected layer. The first two BiLSTM cells have two layers (with 0.1 dropout) with the last cell only having a single layer. Each BiLSTM returns a vector of dimension 128 (for every time-step). The input to the model is ten time-series features, extracted from the original particle coordinates. These were obtained from an extensive literature review and include z-normalized positions, displacements, step sizes, angles, and features that involve cumulative displacements and logarithms of position. We implement data augmentation techniques during training, namely Gaussian noise, random rotations, axial flips and (temporal) trajectory truncation. Training data consisted of 5 million tracks from a broad range of parameters simulated using the AnDi Github package [**?**]. Identical, but separate, models were trained for anomalous exponent (alpha), diffusion coefficient (K) and protein state variable, with the addition of a LogSoftmax layer at the output of the state model (classification task). Each model was trained independently of the others and outputs a time-series, of the same length as the original particle coordinates, providing alpha, K, and state at every time-step (pointwise inference). Alpha and K predicted time-series were smoothed using a threshold-based approach and median filtering. For changepoint detection, we employed the PELT algorithm with an L2 cost function (using ruptures Python library [54]) and combined changepoints from alpha and K. We found this worked better than using all three variables. Models were trained for a maximum of 30 epochs, and the epoch with the lowest validation loss was selected. As each model is small (∼500k trainable parameters), the entire training process can be completed on a single GPU, taking at most ∼10hrs per model.

REFERENCES

[1] P. J. Slator, C. W. Cairo, and N. J. Burroughs, Detection of diffusion heterogeneity in single particle tracking trajectories using a hidden Markov model with measurement noise propagation, PLOS One **10**, e0140759 (2015).

[2] P. J. Slator and N. J. Burroughs, A hidden Markov model for detecting confinement in single-particle tracking trajectories, Biophysical Journal **115**, 1741 (2018).

[3] J. Janczura, M. Balcerek, K. Burnecki, A. Sabri, M. Weiss, and D. Krapf, Identifying heterogeneous diffusion states in the cytoplasm by a hidden Markov model, New Journal of Physics **23**, 053018 (2021).

[4] J. A. Helmuth, C. J. Burckhardt, P. Koumoutsakos, U. F. Greber, and I. F. Sbalzarini, A novel supervised trajectory segmentation algorithm identifies distinct types of human adenovirus motion in host cells, Journal of Structural Biology **159**, 347 (2007).

[5] M. Arts, I. Smal, M. W. Paul, C. Wyman, and E. Meijering, Particle mobility analysis using deep learning and the moment scaling spectrum, Scientific Reports **9**, 1 (2019).

[6] A. Sosa-Costa, I. K. Piechocka, L. Gardini, F. S. Pavone, M. Capitanio, M. F. Garcia-Parajo, and C. Manzo, PLANT: a method for detecting changes of slope in noisy trajectories, Biophysical Journal **114**, 2044 (2018).

[7] D. Montiel, H. Cang, and H. Yang, Quantitative characterization of changes in dynamical behavior for single-particle tracking studies, The Journal of Physical Chemistry B **110**, 19763 (2006).

[8] S. Yin, N. Song, and H. Yang, Detection of velocity and diffusion coefficient change points in single-particle trajectories, Biophysical Journal **115**, 217 (2018).

[9] P. Dosset, P. Rassam, L. Fernandez, C. Espenel, E. Rubinstein, E. Margeat, and P.-E. Milhiet, Automatic detection of diffusion modes within biological membranes using back-propagation neural network, BMC Bioinformatics **17**, 1 (2016).

[10] A. R. Vega, S. A. Freeman, S. Grinstein, and K. Jaqaman, Multistep track segmentation and motion classification for transient mobility analysis, Biophysical Journal **114**, 1018 (2018).

[11] Y. Lanoiselée and D. S. Grebenkov, Unraveling intermittent features in single-particle trajectories by a local convex hull method, Physical Review E **96**, 022144 (2017).

[12] G. Sikora, A. Wyłomańska, J. Gajda, L. Solé, E. J. Akin, M. M. Tamkun, and D. Krapf, Elucidating distinct ion channel populations on the surface of hippocampal neurons via single-particle tracking recurrence analysis, Physical Review E **96**, 062404 (2017).

[13] Y. Matsuda, I. Hanasaki, R. Iwao, H. Yamaguchi, and T. Niimi, Estimation of diffusive states from single-particle trajectory in heterogeneous medium using machine-learning methods, Physical Chemistry Chemical Physics **20**, 24099 (2018).

[14] C. Metzner, C. Mark, J. Steinwachs, L. Lautscham, F. Stadler, and B. Fabry, Superstatistical analysis and modelling of heterogeneous random walks, Nature Communications **6**, 1 (2015).

[15] G. J. Schütz, H. Schindler, and T. Schmidt, Single-molecule microscopy on model membranes reveals anomalous diffusion, Biophysical Journal **73**, 1073 (1997).

[16] R. Simson, E. D. Sheets, and K. Jacobson, Detection of temporary lateral confinement of membrane proteins using single-particle tracking analysis, Biophysical Journal **69**, 989 (1995).

[17] N. Meilhac, L. Le Guyader, L. Salome, and N. Destainville, Detection of confinement and jumps in single-molecule membrane trajectories, Physical Review E **73**, 011915 (2006).

[18] M. Renner, L. Wang, S. Levi, L. Hennekinne, and A. Triller, A simple and powerful analysis of lateral subdiffusion using single particle tracking, Biophysical Journal **113**, 2452 (2017).

[19] Z. Chen, L. Geffroy, and J. S. Biteen, NOBIAS: Analyzing anomalous diffusion in single-molecule tracks with nonparametric Bayesian inference, Frontiers in Bioinformatics , 40 (2021).

[20] S. Matsuoka, T. Shibata, and M. Ueda, Statistical analysis of lateral diffusion and multistate kinetics in single-molecule imaging, Biophysical Journal **97**, 1115 (2009).

[21] C. P. Calderon and K. Bloom, Inferring latent states and refining force estimates via hierarchical Dirichlet process modeling in single particle tracking experiments, PLOS One **10**, e0137633 (2015).

[22] A. Weron, K. Burnecki, E. J. Akin, L. Solé, M. Balcerek, M. M. Tamkun, and D. Krapf, Ergodicity breaking on the neuronal surface emerges from random switching between diffusive states, Scientific Reports **7**, 5404 (2017).

[23] T. Wagner, A. Kroll, C. R. Haramagatti, H.-G. Lipinski, and M. Wiemann, Classification and segmentation of nanoparticle diffusion trajectories in cellular micro environments, PLOS One **12**, e0170165 (2017).

[24] P. J. Bosch, J. S. Kanger, and V. Subramaniam, Classification of dynamical diffusion states in single molecule tracking microscopy, Biophysical Journal **107**, 588 (2014).

[25] F. Persson, M. Lindén, C. Unoson, and J. Elf, Extracting intracellular diffusive states and transition rates from single-molecule tracking data, Nature Methods **10**, 265 (2013).

[26] N. Monnier, Z. Barry, H. Y. Park, K.-C. Su, Z. Katz, B. P. English, A. Dey, K. Pan, I. M. Cheeseman, R. H. Singer, *et al.*, Inferring transient particle transport dynamics in live cells, Nature Methods **12**, 838 (2015).

[27] S. Bo, F. Schmidt, R. Eichhorn, and G. Volpe, Measurement of anomalous diffusion using recurrent neural networks, Physical Review E **100**, 010102 (2019).

[28] B. Requena, S. Masó-Orriols, J. Bertran, M. Lewenstein, C. Manzo, and G. Muñoz-Gil, Inferring pointwise diffusion properties of single trajectories with deep learning, Biophysical Journal **122**, 4360 (2023).

[29] J. Pineda, B. Midtvedt, H. Bachimanchi, S. Noé, D. Midtvedt, G. Volpe, and C. Manzo, Geometric deep learning reveals the spatiotemporal features of microscopic motion, Nature Machine Intelligence **5**, 71 (2023).

[30] H. Kabbech and I. Smal, Identification of diffusive states in tracking applications using unsupervised deep learning methods, in *2022 IEEE 19th International Symposium on Biomedical Imaging (ISBI)* (IEEE, 2022) pp. 1–4.
[31] H. Seckler and R. Metzler, Change-point detection in anomalous-diffusion trajectories utilising machine-learning-based uncertainty estimates, Journal of Physics: Photonics **6**, 045025 (2024).
[32] X. Qu, Y. Hu, W. Cai, Y. Xu, H. Ke, G. Zhu, and Z. Huang, Semantic segmentation of anomalous diffusion using deep convolutional networks, Physical Review Research **6**, 013054 (2024).
[33] D. Ershov, M.-S. Phan, J. W. Pylvänäinen, S. U. Rigaud, L. Le Blanc, A. Charles-Orszag, J. R. Conway, R. F. Laine, N. H. Roy, D. Bonazzi, *et al.*, TrackMate 7: integrating state-of-the-art segmentation algorithms into tracking pipelines, Nature Methods **19**, 829 (2022).
[34] B. Midtvedt, S. Helgadottir, A. Argun, J. Pineda, D. Midtvedt, and G. Volpe, Quantitative digital microscopy with deep learning, Applied Physics Reviews **8**, 011310 (2021).
[35] D. B. Allan, T. Caswell, N. C. Keim, C. M. van der Wel, and R. W. Verweij, Trackpy: Fast, flexible particle-tracking toolkit (2024).
[36] J. C. Crocker and D. G. Grier, Methods of digital video microscopy for colloidal studies, Journal of Colloid and Interface Science **179**, 298 (1996).
[37] D. Li, Q. Yao, and Z. Huang, Wavenet-based deep neural networks for the characterization of anomalous diffusion (wadnet), Journal of Physics A: Mathematical and Theoretical **54**, 404003 (2021).
[38] S. Hochreiter, Long short-term memory, Neural Computation MIT-Press (1997).
[39] A. Vaswani, Attention is all you need, Advances in Neural Information Processing Systems (2017).
[40] S. Arlot, A. Celisse, and Z. Harchaoui, A kernel multiple change-point algorithm via model selection, Journal of machine learning research **20**, 1 (2019).
[41] C. M. Bishop and N. M. Nasrabadi, *Pattern recognition and machine learning*, Vol. 4 (Springer, 2006).
[42] G. Schwarz, Estimating the dimension of a model, The Annals of Statistics , 461 (1978).
[43] H. Buena-Maizón and F. J. Barrantes, A deep learning-based approach to model anomalous diffusion of membrane proteins: the case of the nicotinic acetylcholine receptor, Briefings in Bioinformatics **23**, 1 (2022).
[44] N. Granik, L. E. Weiss, E. Nehme, M. Levin, M. Chein, E. Perlson, Y. Roichman, and Y. Shechtman, Single-particle diffusion characterization by deep learning, Biophysical Journal **117**, 185 (2019).
[45] L. Loeff, J. W. J. Kerssemakers, C. Joo, and C. Dekker, Autostepfinder: A fast and automated step detection method for single-molecule analysis, Patterns **2**, 100256 (2021).
[46] R. Killick, P. Fearnhead, and I. A. Eckley, Optimal detection of changepoints with a linear computational cost, Journal of the American Statistical Association **107**, 1590 (2012).
[47] S. Lloyd, Least squares quantization in pcm, IEEE transactions on information theory **28**, 129 (1982).
[48] S. Asghar, R. Ni, and G. Volpe, U-net 3+ for anomalous diffusion analysis enhanced with mixture estimates (u-and-me) in particle-tracking data, arXiv preprint arXiv:2502.19253 (2025).
[49] H. Huang, L. Lin, R. Tong, H. Hu, Q. Zhang, Y. Iwamoto, X. Han, Y.-W. Chen, and J. Wu, Unet 3+: A full-scale connected unet for medical image segmentation, in *ICASSP 2020-2020 IEEE international conference on acoustics, speech and signal processing (ICASSP)* (IEEE, 2020) pp. 1055–1059.
[50] Y. Ahsini, M. Escoto, and J. A. Conejero, Anomalousnet: A hybrid approach with attention u-nets and change point detection for accurate characterization of anomalous diffusion in video data, arXiv preprint arXiv:2504.05271 (2025).
[51] O. Ronneberger, P. Fischer, and T. Brox, U-net: Convolutional networks for biomedical image segmentation, in *Medical image computing and computer-assisted intervention–MICCAI 2015: 18th international conference, Munich, Germany, October 5-9, 2015, proceedings, part III 18* (Springer, 2015) pp. 234–241.
[52] R. B. Tokime, X. Maldague, and L. Perron, Automatic defect detection for x-ray inspection: A u-net approach for defect segmentation, Proceedings of the Digital Imaging and Ultrasonics for NDT (2019).
[53] O. Oktay, J. Schlemper, L. L. Folgoc, M. Lee, M. Heinrich, K. Misawa, K. Mori, S. McDonagh, N. Y. Hammerla, B. Kainz, *et al.*, Attention u-net: Learning where to look for the pancreas, arXiv preprint arXiv:1804.03999 (2018).
[54] C. Truong, L. Oudre, and N. Vayatis, Selective review of offline change point detection methods, Signal Processing **167**, 107299 (2020).
[55] J. Kæstel-Hansen, M. de Sautu, A. Saminathan, G. Scanavachi, R. F. B. D. C. Correia, A. J. Nielsen, S. V. Bleshøy, W. Boomsma, T. Kirchhausen, and N. S. Hatzakis, Deep learning assisted single particle tracking for automated correlation between diffusion and function, bioRxiv (2023).
[56] J. Bae, Y. Baek, and H. Jeong, Exploring how deep learning decodes anomalous diffusion via grad-cam, arXiv preprint arXiv:2410.16345 (2024).
[57] J. Park, N. Sokolovska, C. Cabriel, I. Izeddin, and J. Miné-Hattab, Freetrace (2024).
[58] X. Shi, Z. Chen, H. Wang, D.-Y. Yeung, W. kin Wong, and W. chun Woo, Convolutional lstm network: A machine learning approach for precipitation nowcasting (2015), arXiv:1506.04214 [cs.CV].
[59] J. Park, N. Sokolovska, C. Cabriel, I. Izeddin, and J. Miné-Hattab, Bi-add (2024).
