# Supplementary material for: Quantitative evaluation of methods to analyze motion changes in single-particle experiments
Source: Nat Commun. 2025 Jul 22;16:6749. doi: 10.1038/s41467-025-61949-x (PMC12283970; doi:10.1038/s41467-025-61949-x)
Supplement: Supplementary file 2 — Reporting Summary [file 41467_2025_61949_MOESM2_ESM.pdf]

nature portfolio

Corresponding author(s): Muñoz-Gil, Volpe, Manzo

Last updated by author(s): Jun 19, 2025

Reporting Summary

Nature Portfolio wishes to improve the reproducibility of the work that we publish. This form provides structure for consistency and transparency in reporting. For further information on Nature Portfolio policies, see our Editorial Policies and the Editorial Policy Checklist.

Statistics

For all statistical analyses, confirm that the following items are present in the figure legend, table legend, main text, or Methods section.

n/a	Confirmed	
x	☐	The exact sample size (*n*) for each experimental group/condition, given as a discrete number and unit of measurement
x	☐	A statement on whether measurements were taken from distinct samples or whether the same sample was measured repeatedly
x	☐	The statistical test(s) used AND whether they are one- or two-sided<br>*Only common tests should be described solely by name; describe more complex techniques in the Methods section.*
x	☐	A description of all covariates tested
x	☐	A description of any assumptions or corrections, such as tests of normality and adjustment for multiple comparisons
x	☐	A full description of the statistical parameters including central tendency (e.g. means) or other basic estimates (e.g. regression coefficient) AND variation (e.g. standard deviation) or associated estimates of uncertainty (e.g. confidence intervals)
x	☐	For null hypothesis testing, the test statistic (e.g. *F*, *t*, *r*) with confidence intervals, effect sizes, degrees of freedom and *P* value noted<br>*Give P values as exact values whenever suitable.*
x	☐	For Bayesian analysis, information on the choice of priors and Markov chain Monte Carlo settings
x	☐	For hierarchical and complex designs, identification of the appropriate level for tests and full reporting of outcomes
x	☐	Estimates of effect sizes (e.g. Cohen's *d*, Pearson's *r*), indicating how they were calculated

Our web collection on statistics for biologists contains articles on many of the points above.

Software and code

Policy information about availability of computer code

Data collection	The labeled benchmark dataset used in this study is available on Zenodo (https://doi.org/10.5281/zenodo.14281478).<br>All datasets generated for the Challenge can be accessed on the Codalab platform (https://codalab.lisn.upsaclay.fr/competitions/16618}<br>(registration required).
Data analysis	All code used to generate the Challenge datasets is publicly available via the andi_datasets repository on GitHub: https://github.com/AnDiChallenge/andi_datasets and on Zenodo (https://doi.org/10.5281/zenodo.10259556).

For manuscripts utilizing custom algorithms or software that are central to the research but not yet described in published literature, software must be made available to editors and reviewers. We strongly encourage code deposition in a community repository (e.g. GitHub). See the Nature Portfolio guidelines for submitting code & software for further information.

Data

Policy information about availability of data

All manuscripts must include a data availability statement. This statement should provide the following information, where applicable:

- Accession codes, unique identifiers, or web links for publicly available datasets
- A description of any restrictions on data availability
- For clinical datasets or third party data, please ensure that the statement adheres to our policy

Provide your data availability statement here.

Research involving human participants, their data, or biological material

Policy information about studies with human participants or human data. See also policy information about sex, gender (identity/presentation), and sexual orientation and race, ethnicity and racism.

Reporting on sex and gender	Not applicable
Reporting on race, ethnicity, or other socially relevant groupings	Not applicable
Population characteristics	Not applicable
Recruitment	Not applicable
Ethics oversight	Not applicable

Note that full information on the approval of the study protocol must also be provided in the manuscript.

Field-specific reporting

Please select the one below that is the best fit for your research. If you are not sure, read the appropriate sections before making your selection.

☒ Life sciences ☐ Behavioural & social sciences ☐ Ecological, evolutionary & environmental sciences

For a reference copy of the document with all sections, see nature.com/documents/nr-reporting-summary-flat.pdf

Life sciences study design

All studies must disclose on these points even when the disclosure is negative.

Sample size	The number of datasets was chosen to reflect realistic experimental throughput: for each biological scenario ("experiment"), we simulated 30 fields of view, which we group conceptually as three technical replicates of ten videos each. In practice, our design mirrors the data volume and variability encountered in typical live-cell single-particle tracking studies. By providing 30 independent views per scenario, we ensure robust estimates of parameters while keeping the computational load manageable; this balance between realism and statistical power underpins the sufficiency of our dataset for benchmarking analysis methods. Further details on dataset structure and rationale are given in the main text.
Data exclusions	As described in the manuscript, three experiments used in the challenge contained parameters meant to assess potential biases of the methods and although they were used for challenge ranking, they were not discussed in the article.
Replication	Our datasets are wholly synthetic—trajectory and video simulations drawn from known parameter distributions— so traditional technical replication does not apply. Instead, we capture variability by generating multiple independent realizations for each scenario. Specifically, we simulate 30 fields of view per condition.<br>Our datasets are wholly synthetic—trajectory and video simulations drawn from known parameter distributions— so traditional technical replication does not apply. Instead, we capture variability by generating multiple independent realizations for each scenario. Specifically, we simulate 30 fields of view per condition.
Randomization	Not applicable
Blinding	Challenge participants did not have access to the ground truth data.

Reporting for specific materials, systems and methods

We require information from authors about some types of materials, experimental systems and methods used in many studies. Here, indicate whether each material, system or method listed is relevant to your study. If you are not sure if a list item applies to your research, read the appropriate section before selecting a response.

Materials & experimental systems

n/a	Involved in the study
☒	☐ Antibodies
☒	☐ Eukaryotic cell lines
☒	☐ Palaeontology and archaeology
☒	☐ Animals and other organisms
☒	☐ Clinical data
☒	☐ Dual use research of concern
☒	☐ Plants

Methods

n/a	Involved in the study
☒	☐ ChIP-seq
☒	☐ Flow cytometry
☒	☐ MRI-based neuroimaging

Plants

Seed stocks	Not applicable
Novel plant genotypes	Not applicable
Authentication	Not applicable
